# Distinct bacterial signature in the raw coal with different heating value

**DOI:** 10.3389/fmicb.2024.1459596

**Published:** 2024-09-05

**Authors:** Haijiang Zou, Miaomiao Tian, Jianmin Xu, Guowei Li, Hui Chen, Junjun Yang, Pengtao Ling, Zhenxing Shen, Siyu Guo

**Affiliations:** ^1^Department of Environmental Science and Engineering, Xi’an Jiaotong University, Xi’an, China; ^2^Shaanxi Coalbed Methane Development Co., Shaanxi Coal and Chemical Industry Group Co., Ltd., Xi’an, Shaanxi, China; ^3^Center for Mitochondrial Biology and Medicine, The Key Laboratory of Biomedical Information Engineering of Ministry of Education, School of Life Science and Technology, Xi’an Jiaotong University, Xi’an, Shaanxi, China; ^4^Shaanxi Coal Industry Company Limited, Xi’an, Shaanxi, China; ^5^Xijing Hospital of Digestive Diseases, Xijing Hospital of Air Force Military Medical University, Xi'an, China; ^6^Department of Civil Engineering, Xi’an Jiaotong University, Xi’an, China; ^7^School of Civil Engineering and Architecture, Henan University of Science and Technology, Luoyang, China

**Keywords:** coal quality, bacterial community, co-occurrence network, heating value, *Anaerostipes*

## Abstract

**Introduction:**

Coal represents a significant natural resource in our world, and its quality and commercial value is primarily determined by its heating capacity. Numerous scientists worldwide have attempted to explore the impact of various environmental factors on coal rank, yet their conclusions are often inconsistent.

**Methods:**

In this study, the Illumina MiSeq sequencing approach was used to analyze the bacterial community from a low-rank coal mine as well as a high-rank mine. Moreover, we investigated the relationship between the physical and chemical properties of the coal and the bacterial composition.

**Results:**

Overall, we found that the high-rank coal exhibited higher heating value but higher total sulfur and lead levels. Considering the community of bacteria, the abundances of *Phascolarctobacterium* and *Anaerostipes* were highly elevated in the high-rank coal group. Most interestingly, the *Anaerostipes* abundance was correlated with coal quality positively. Additionally, the co-occurrence network of the bacterial community in the high-rank coal group showed much higher complexity. The bacterial functional potential predictions indicated elevated levels of phosphoenolpyruvate carboxykinase ATP, succinate dehydrogenase fumarate reductase flavoprotein subunit, and methylenetetrahydrofolate dehydrogenase NADP methenyltetrahydrofolate cyclohydrolase pathways.

**Conclusion:**

This study revealed that high-rank coal had more complicated co-occurrence network and elevated *Anaerostipes* abundance, which may suggest a potential biological pathway that can be explored to enhance coal quality.

## 1 Introduction

During the recent decades, there has been a significant increase in global energy consumption, with over 40% of the growth being generated by India and China (Tollefson, 2017). Currently, coal is the dominant energy source in China due to its abundant reserves. As the world’s primary resource, coal is primarily evaluated based on its calorific capacity when determining its quality. High-quality coal resources are typically characterized by high heating capacity and are thus highly desirable. Conversely, low-quality coal with lower heating capacity has remained a challenge for scientists seeking to utilize and convert it into a better energy source (Park et al., 2021; Thomas and Fariborz, 2000).

The coal is a complex mixture where parameters such as volatile matter, moisture, sulfur, and ash have complicated effects on the coal quality. To address these challenges, scientists worldwide have conducted extensive research to make coal clean and safe for use. They have made every effort to investigate the effects of various properties on coal rank using both experimental and numerical simulation methods, but the findings are ambiguous (Borowski and Ozga, 2020; Li S. et al., 2020; Zhai et al., 2022; Zhou et al., 2020).

Recent studies have implied that the bacterial community may be related to the coal quality. For example, there were some studies explored the function and diversity of bacterial community in biogenic coal-bed methane with different coal ranks (Guo et al., 2012). Robbins et al. (2016) found a significant negative correlation between rank and bacterial diversity, that is, bacterial diversity decreased significantly as rank maturity increased. These studies provide primary evidences linking the bacterial community to the coal rank.

The bacterial diversity contribute significantly to ecosystem structure due to their high diversity and variability, where subsurface bacterial communities degraded coal components to form coal properties (Wang et al., 2019). Further study has shown that the bacteria are responsible for the formation of coal and methane in coal mines, as they consume coal methoxyl and also produce methane (Lloyd et al., 2021). The fixed carbon content is a key indicator of coal rank, and the bacteria are considered to be involved in carbon-fixing process. Several studies have established that Proteobacteria played important roles in eutrophication, since it is linked to high carbon utilization and critical to the carbon cycle (Xu et al., 2023).

Additionally, it has been widely accepted that the coal quality is mainly affected by physical and chemical factors during the coal formation stage. However, the bacterial community are also involved in this process. The biotransformation of coal by methanogenic microorganisms is bound to cause changes in the physical chemistry properties of coal, which may ultimately led to altered coal quality (Dong et al., 2024). All these studies suggest that the bacterial community may be also involved in coal ranking.

In this study, coal samples were collected in a representative coal mine with relative higher heating value and a representative coal mine with lower heating value, respectively. The objective of this study was to examine the relationship between the bacterial community structure and coal quality. To achieve this, the industrial parameters, several heavy metals, and the characteristics of the bacterial community were tested in the coal samples. Additionally, the interaction between the coal matrix, heavy metals, and bacterial community were also analyzed. This study had the following specific objectives: (1) characterize the heavy metal and bacterial profiles in coal samples from different rank coal mines; (2) explore the potential relationships between environmental parameters and the bacterial community in the coal samples; (3) determine the key factors that influence coal rank.

## 2 Methods

### 2.1 Coal samples collection

The study site is located in an area with abundant coal resources. Sampling was conducted in two separate mining areas located in the western part of Xianyang city, Shaanxi province. The coal seams from these two coal mines have mid-high heating values and low-mid ash contents, low-mid sulfur contents, mid-high volatile matter contents, extremely low phosphorus contents, and rich oil content. These coals can be used for power generation, gasification, liquefaction, coal water slurry coal, and other purposes.

The heating value is a key indicator to evaluate the coal quality, then we employed the heating value to group the samples. A total of 23 samples of freshly extracted coal were collected from coal mines by the professionals. The coal samples (*n* = 10) from the Mengcun mining area located in Changwu district were identified to have lower heating values and assigned to the low-rank coal group, while the coal samples (*n* = 13) from the Xiaozhuang mining area located in Binzhou district were identified to have higher heating values and assigned to the high-rank coal group.

All the coal samples selected were prepared as the following steps. Firstly, the coal samples were taken by the professionals through using the core drilling machine (XY-6B, Wuxi Prospecting Machinery General Factory), then divided into two separate parts and stored in airtight containers. One part was for physicochemical properties and elemental analysis, and the other one part was for bacterial community analysis. Secondly, all the samples were processed as small blocks (smaller than 2cm^3^) and then crushed to a particle size of less than 0.5 mm and then further analyses following the Chinese standard GB 474–2008. To ensure that the coal samples did not become contaminated with other bacteria in the environment, the samples were prepared in an anaerobic and aseptic chamber by using a bur-style coffee grinder and then a mortar and pestle. Thirdly, the samples used for bacterial community test were frozen using liquid nitrogen and stored in sterile EP tubes at −80°C.

### 2.2 Physicochemical properties and elemental analyses

The heating value was evaluated following the Chinese standard GB/T 213–2008: A certain amount of every sample (0.9 g-1.1 g) is completely burned in an airtight vessel (oxygen bomb) under the condition of the existence of excess oxygen. The heat released from the burning coal is absorbed by a certain amount of water, and then the rise in water temperature is used to calculate the heating value of the sample. The samples were analyzed based on the Chinese standard GB/T 212–2008 for basic parameters such as water, ash, and volatile matter. The samples were also analyzed according to the Chinese standard GB/T 214–2007 for total sulfur, Chinese standard GB/T 3558–2014 for chlorine content and Chinese standard GB/T 4633–2014 for phosphorus content. Additionally, the samples were tested by X-ray diffraction in the low-temperature ash residues for heavy metals concentrations as described (Saha and Roychowdhury, 2023). The methane concentrations were measured by using Shimadzu GC-2014 gas chromatograph (Shimadzu, Japan) following the instructions.

### 2.3 DNA extraction of the coal samples and sequencing

We extracted DNA from coal samples following the manufacturer’s protocol using a DNA Extraction Kit. A NanoDrop instrument was used for quality verification of the extracted DNA. For further processing, DNA was diluted to 1 ng/μL and stored at −20°C. The V3 and V4 variable regions of the 16S rRNA gene were amplified by using universal primers. Agencourt AMPure XP beads were employed to purify the amplicons, and a Qubit dsDNA assay kit was used to quantify them. In order to sequence the purified amplicons, equal amounts were pooled. Using the Illumina Miseq platform, the sequencing analysis was conducted.

### 2.4 Bioinformatics and statistical analysis of the sequencing data

In accordance with Zhang’s et al. (2016) description, raw sequencing data was quality-controlled. To determine representative sequences, quality-controlled sequences were clustered and sorted by decreasing abundance using UPARSE. During this process, singletons were omitted. UPARSE was used to remove chimeric sequences from OTUs and classify them based on 97% similarity. Using the RDP Classifier, every sequence of the 16S rRNA gene was compared with the RDP database using a 70% confidence threshold. In order to determine the diversity of samples, QIIME calculated a nonparametric Shannon-Wiener diversity index. To visualize UniFrac dissimilarity. For detecting differential abundances between two groups, Lefse was employed. The PCoA plots and all bars were generated with R. A network analysis was conducted based on the bacteria found in at least half of the samples as described previously (Sun et al., 2019). The correlation networks were constructed in Gephi software by using robust correlations.

### 2.5 Statistical analysis

In this study, IBM SPSS version 19.0 was used to analyze the data. Means and standard errors of the means (SEMs) were calculated. The difference between the two groups was determined by an independent samples *t*-test. A *p*-value of less than 0.05 was considered statistically significant.

## 3 Results

### 3.1 Coal-rank related parameters

Multiple sites within the mining area were sampled to eliminate the effects of other factors, including the climate, mining area scale and management. By using this approach, mining areas can be characterized in terms of the diversity of their bacterial communities. All samples were collected from the sites indicated in Supplementary Figure S1.

**Figure 1:**
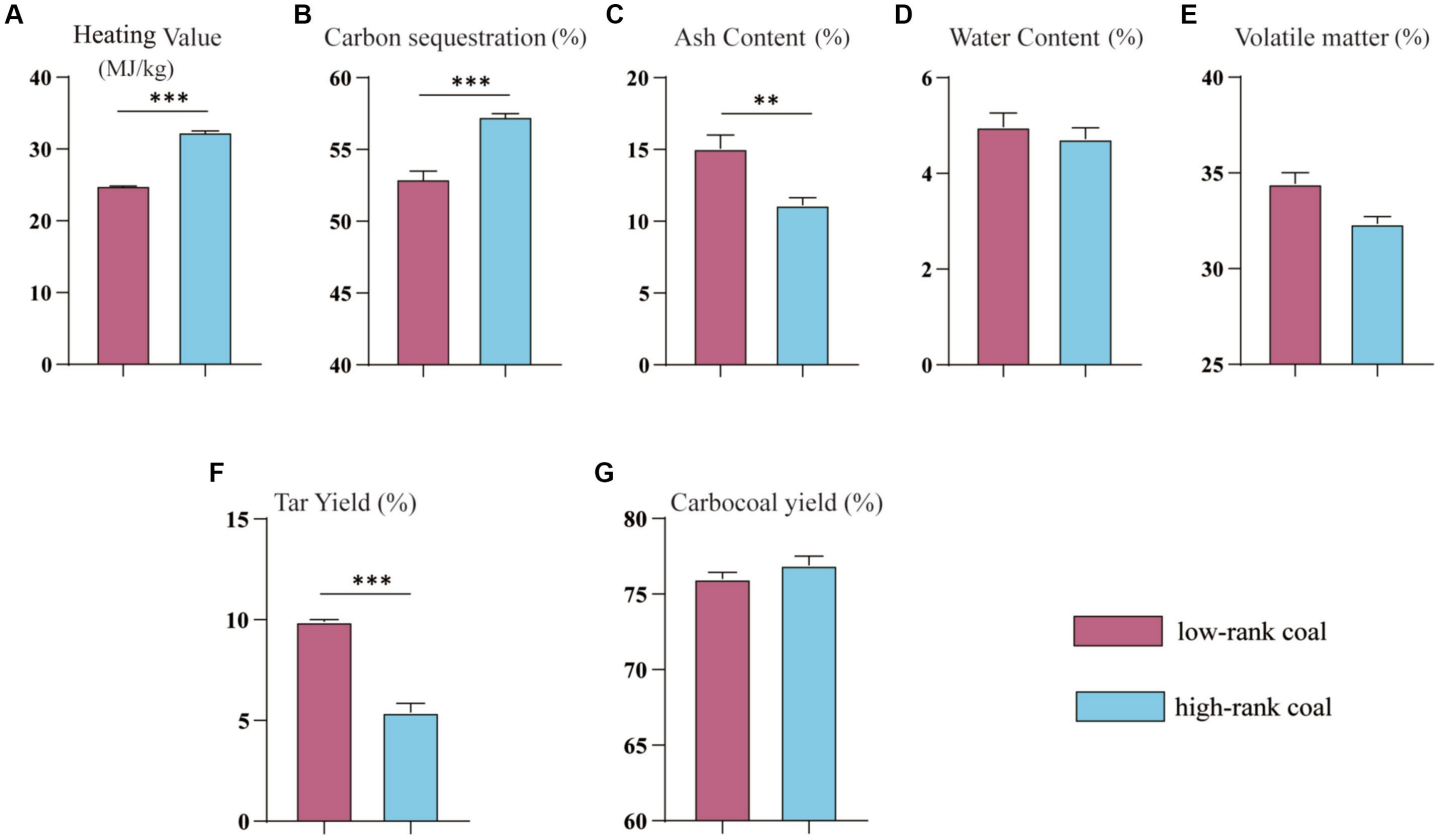
Heating value, Tar Yield and other coal rank related parameters between the two groups. **(A)** Heating Value. **(B)** Carbon sequestration. **(C)** Ash content. **(D)** Water content. **(E)** Volatile matter. **(F)** Tar Yield. **(G)** Carbocoal yield. *p < 0.05.

The heating value and tar yield of the collected samples were shown in Figure 1. The samples in the high-rank coal group had a higher heating value (Figure 1A), higher carbon sequestration capacity, and lower ash content compared with the samples in the low-rank coal group (Figures 1B,C), *p* < 0.05. Both groups had similar water and volatile matter contents (Figures 1D,E), *p* > 0.05. However, the tar yield in the high-rank coal group was much lower compared with the low-rank coal group (Figure 1F), *p* < 0.05, although they have similar carbocoal yield levels (Figure 1G), *p* > 0.05. There is no doubt that the physicochemical properties of coal are dependent on coal rank, and with the increase of metamorphism degree there is a tendency for heating value to rise (Li X. et al., 2020). However, all these data showed that there is no obvious relationship between heating value and coal metamorphism degree, and they may be also affected by complex physicochemical properties and other environmental factors.

### 3.2 Chemical elements and heavy metal contents

To further explore the potential factors affecting coal rank, the concentrations of certain chemical elements in the raw coal, including total sulfur, fluorine, phosphorus, and chlorine were determined and are shown in Supplementary Figure S2A. In terms of the environmental pollutants in the raw coal, compared with the low-rank coal, the high-rank coal samples contained more sulfur, *p* < 0.05; Additionally, the heavy metals, including vanadium, gallium, thorium, uranium, germanium, arsenic, and lead were also determined and are shown in Supplementary Figure S2B. There was a higher concentration of lead in the high rank coal group, *p* < 0.05; The other heavy metals, however, did not differ between the groups, *p* > 0.05. Similarly, also in the clean coal, there were no significant differences in the concentrations of chemical elements and heavy metals between the two groups, *p* > 0.05; however, the total sulfur content was higher and methane level was lower in the high-rank coal than those in the low-rank coal group, *p* < 0.05, as shown in Supplementary Figure S2C. Considering the concentrations of chemical elements and heavy metals, the results suggest that coal rank is more dependent on other environmental factors.

### 3.3 Bacterial community analysis

**Figure 2:**
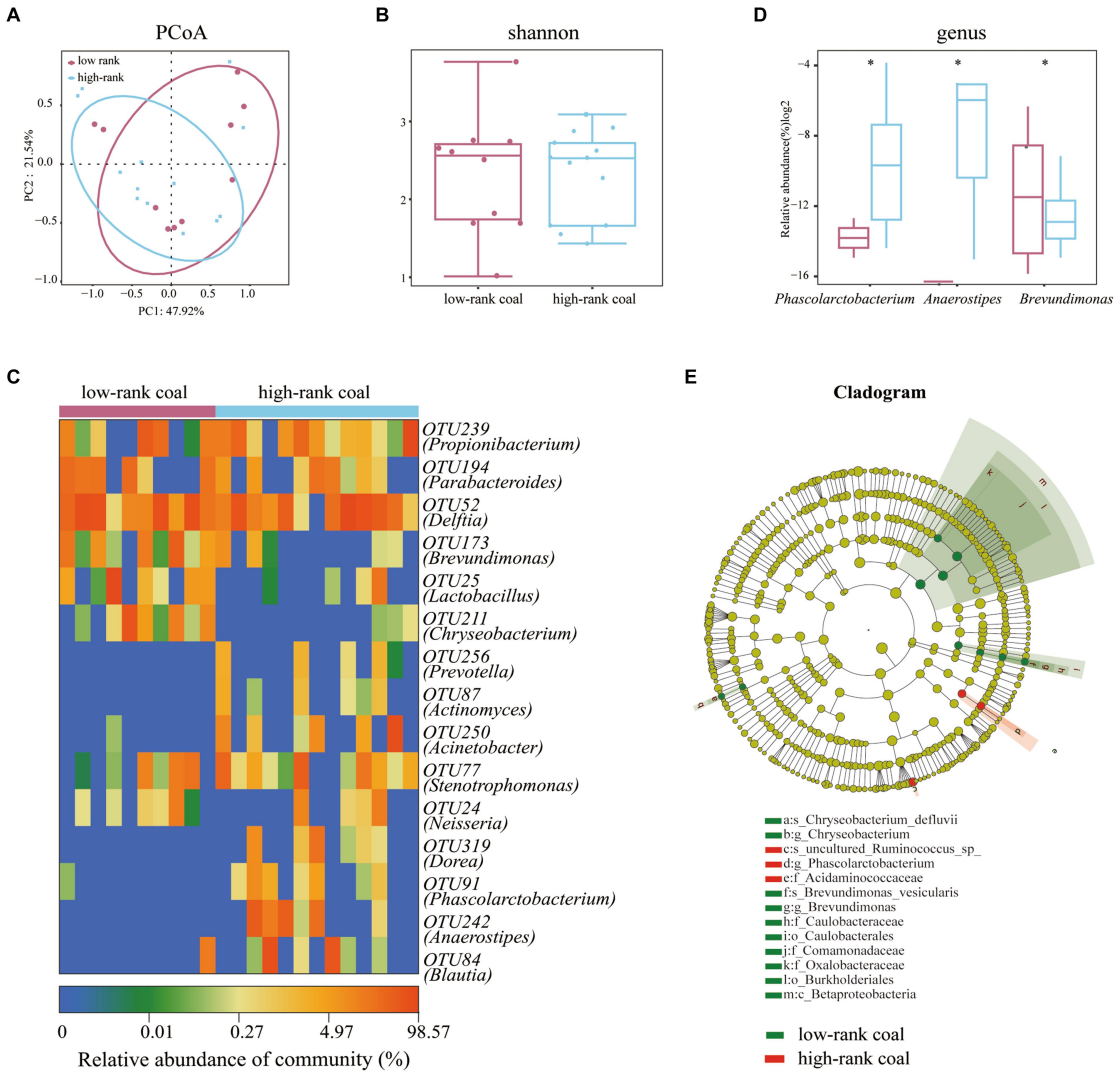
Bacterial community analysis of the two groups. **(A)** PCoA analysis plots of Bray–Curtis dissimilarity between the two groups. **(B)** Shannon diversity scores. **(C)** Heatmap of key OTUs. **(D)** Altered bacterial community with different abundance at the genus level. **(E)** Lefse analysis of bacterial abundance between the two groups. *n* = 10 in the low-rank coal group; *n* = 13 in the high-rank coal group.

Methane levels in coal samples are influenced by the bacterial community, and considering the diversity of methane between the two groups, we sought to explore the bacterial community characteristics furtherly. In terms of bacterial composition, the Principle Coordinates Analysis (PCoA) plot showed no significant difference between the two groups (Figure 2A). Similarly, the two groups did not differ significantly in terms of bacterial diversity based on the Shannon index (Figure 2B). Heatmaps of the key OTUs revealed that there were some differences between the two groups, including the abundance of the OUT173 (*Brevundimonas*), OTU211 (*Chryseobacterium*), OTU91 (*Phascolarctobacterium*), and 12 other OTUs (Figure 2C).

To further observe the divergence in the bacterial community, we compared the relative abundance of all the bacterial genus, we found that the relative abundance of *Phascolarctobacterium* and *Anaerostipes* were largely higher in the high-rank coal group, but the relative abundance of *Brevundimonas* was lower in the high-rank coal group, *p* < 0.05 (Figure 2D). According to the linear discriminant analysis of effect size (Lefse) analysis, bacterial taxa with higher abundances in the high-rank coal group primarily originated from the family of Acidaminococcaceae, in contrast, the lower abundance bacteria mainly originated from the class of Betaproteobacteria (Figure 2E).

### 3.4 Micro-ecological network feature of the environmental bacteria

**Figure 3:**
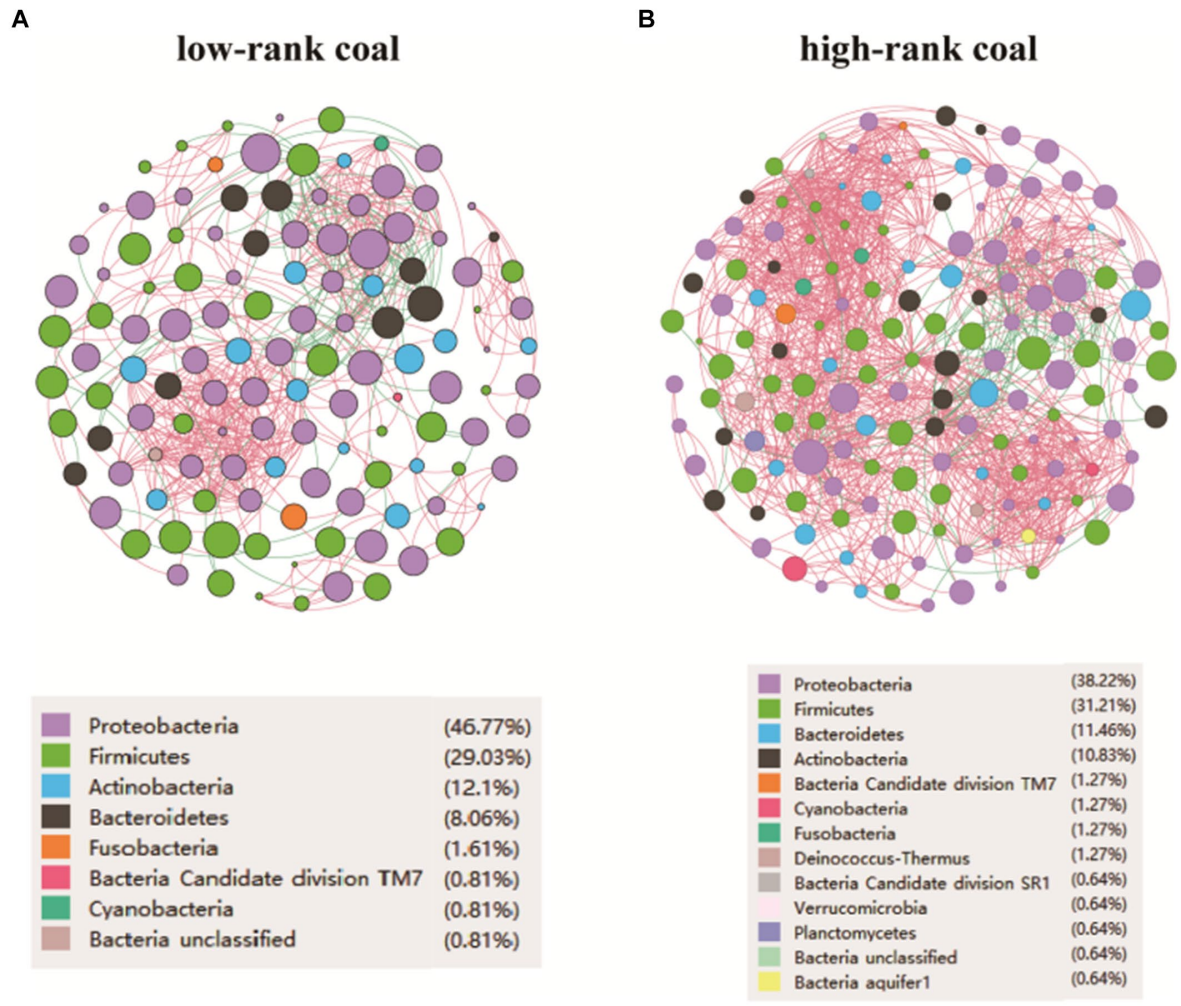
Micro-ecological network feature of the environmental bacteria. **(A)** Network analysis at the genus level in the low-rank coal group. **(B)** Network analysis at the genus level in the high-rank coal group. V, number of nodes. E, number of edges.

A bacterial co-occurrence network can provide insight into patterns and relationships among bacteria. In order to identify how bacteria from the different groups co-occurred, the genus-level network of the two groups were constructed. The networks showed that the high-rank coal group had a more complicated micro-ecological network (V/E = 157/1308) compared with the low-rank coal group (V/E = 124/559), as shown in Figures 3A,B. As the heating value of the samples increased in the high-rank coal group, the network of bacterial interactions showed an increasing trend. It is the first study to apply a symbiotic network of coal bacterial community to understand symbiotic relationships in different rank coal.

### 3.5 Interaction between bacterial community and physicochemical parameters

**Figure 4:**
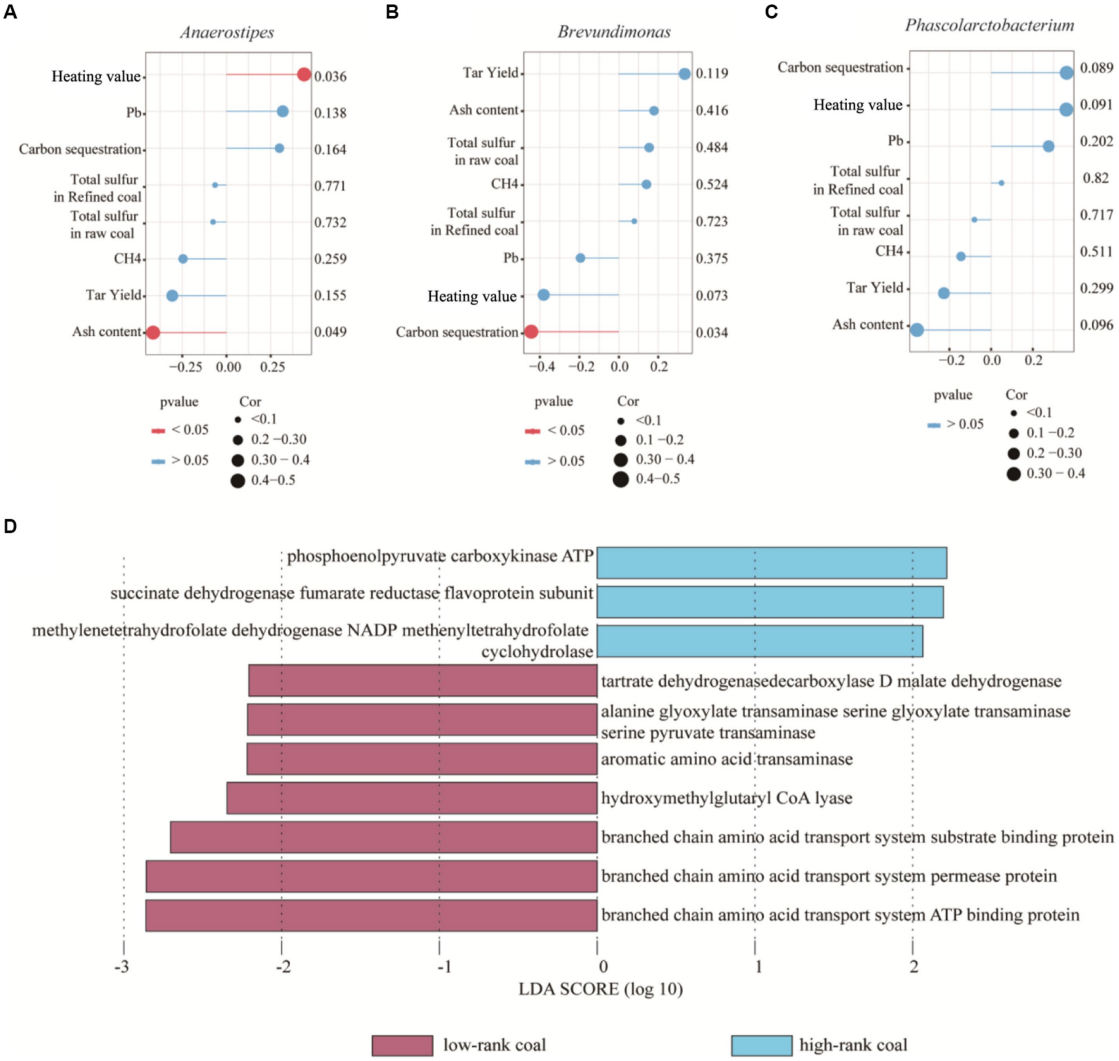
Interaction between bacterial community and physicochemical parameters. **(A)** Relationship between *Anaerostipes* and given indicators. **(B)** Relationship between *Brevundimonasand* and given indicators. **(C)** Relationship between *Phascolarctobacterium* and given indicators. **(D)** Functional divergence between bacteria of two groups using Lefse analysis.

To identify correlations between the environmental bacteria with different abundance and given indicators, including heating value, carbon sequestration, ash content, total sulfur in raw coal and refined coal, CH_4_ and tar yield. Most importantly, there was a positive correlation between *Anaerostipes* abundance and heating value, but a negative correlation between *Anaerostipes* abundance and ash content (*p* < 0.05), but not between any other indicators (*p* > 0.05), as shown in Figure 4A; also, the *Brevundimonas* abundance was negatively correlated with the ash content (*p* < 0.05), indicating that *Anaerostipes* may be potentially key factors improving the coal quality, as shown in Figure 4B. However, no significant relationship was found between the *Phascolarctobacterium* and given indicators, *p* > 0.05, as shown in Figure 4C.

Afterwards, bacteria may play their roles in coal formation through the functional genes located in the bacterial DNA. Based on phenotypic and gene function predictions, different bacteria exhibited similar functions and phenotypes. PICRUSt is a database that matches the metabolism of prokaryotic microorganisms and is widely used to predict the functions of environmental bacteria. We found that the genes related to phosphoenolpyruvate carboxykinase ATP, succinate dehydrogenase fumarate reductase flavoprotein subunit and methylenetetrahydrofolate dehydrogenase NADP methenyltetrahydrofolate cyclohydrolase showed high abundance in the high-rank coal group, suggesting their roles in carbon circulation and energy storage, as shown in Figure 4D. These metabolic pathways play important roles in cell synthesis, decomposition and metabolism. Therefore, the increased metabolism pathway might be another mechanism of energy immobilization.

## 4 Discussion

The physicochemical parameters were commonly regarded to be key indicators of environmental quality, including the soil, various water bodies, rocks and oils, as they can shape the environmental structure. In addition to mediating ecosystem functioning through biogeochemical processes, the bacterial community has been found to be very important to ecosystem stability. Recently, it has been found that plant matter may be transformed into coal on geologic time scales by deep biosphere communities, and methoxyl abundance may affect coal-bed methane production. This study found that the bacterial community varied in the bacterial community and eco-system network between the different rank coal groups. Through this perspective, we can improve the coal quality and uncover the role of bacteria in coal formation.

Environmental factors have been documented to influence bacterial community composition and diversity. It has been shown that coal mine drainage can reshape the complex biochemical processes and then the bacterial community in the coal environment (Zhou et al., 2023). Meanwhile, other studies reported that surface mining activities had led to deterioration of local and regional environmental quality, among which the metals Na and B had strong negative impact on main bacterial profiles. Conversely, the PH, Ca, K, and Fe concentrations were positively correlated with bacterial composition (Hamidovic et al., 2020). In addition, several studies have explored the connection of the heavy metal contents to the bacterial abundance in soils. Although these studies have recognized the close relationship between environmental factors and coal formation, they have mainly focused on the impacts of coal processing on the environment. Nevertheless, whether the bacterial community affects coal quality has received little focused investigation.

In this study, the *Phascolarctobacterium* and *Anaerostipes* were significantly increased. It has been widely accepted that the physicochemical parameters are key indicators affecting coal quality by shaping the environmental structure. Most importantly, the *Phascolarctobacterium* (Shigeno et al., 2019; Watanabe et al., 2012; Wu et al., 2017) and *Anaerostipes* (Bui et al., 2014; Kadowaki et al., 2023; Lee et al., 2021; Morinaga et al., 2021) have been reported that could produce short-chain fatty acids, also considering their roles in shaping the environment by affecting the PH value, it may provide new understanding about the roles of bacterial community in coal formation.

Conversely, the *Brevundimonas* was found to be increased in the low-rank coal group. *Brevundimonas* is a type of Gram-negative bacterium that can live in various environments, including soils, deep subsea floor sediment, purified water and also from the condensation water (Ryan and Pembroke, 2018). However, its roles in the formation of coal environment are not clear and need to be studied furtherly. Considering its relationship with coal quality, strategies targeting the growth and reproduction of *Brevundimonas* may also be implied to improve coal quality.

The bacteria can play their roles through tightly organized network. It is generally believed that higher bacterial richness and network stability are signs of a better and healthier environment. In this study, we found that in the bacterial community interaction network between different types of samples, the Proteobacteria plays crucial roles in keeping it stable. The Proteobacteria has a wider niche and a greater ability to resist environmental interference, making it an ideal bacterium for enhancing bacterial resistance to stress. Increasing network complexity increases the stability of mixed and interactive bacterial community, also enhances resource transfer efficiency. Biological networks are considered stable when they are highly interconnected and complex. Having a high diversity of bacteria increases the likelihood of establishing relationship with nearby bacteria (Pan et al., 2021). The observed phenomenon could be explained by the fact that the more complex micro-ecological network may shape the environment and promote the improvement of coal quality.

Bacteria were found to have similar functions and phenotypes based on their phenotypic and gene function predictions. We found that the genes related to phosphoenolpyruvate carboxykinase ATP, succinate dehydrogenase fumarate reductase flavoprotein subunit and methylenetetrahydrofolate dehydrogenase NADP methenyltetrahydrofolate cyclohydrolase showed high abundance in the high-rank coal group. These metabolic pathways play important roles in cell synthesis, decomposition, and metabolism. Hence, the increased metabolism pathways might be other mechanisms of energy immobilization.

There are several limitations of this study. Firstly, we selected dozens of coal samples to represent the characteristics of the coal mine, but the sample size is smaller to some extent, and the sample size still needs to be further expanded. Secondly, in this study, altered bacterial communities were observed, but whether they were causal or not is unknown, thus, it is necessary to elucidate their roles in energy immobilization. Thirdly, the results we observed need to be confirmed in other regions. Thirdly, there are many indicators to evaluate the quality of coal, but we only used coal heating value to group the samples in this study as all these indicators may be inconsistent. It is also necessary to consider the relationship between these indicators and the bacterial community.

## 5 Conclusion

The physicochemical parameters are commonly regarded as key indicators affecting coal quality by shaping the environmental structure. According to documented evidence, it has been implied the bacterial community may also play synergic roles in the process of coal formation. As a result of the present study, the high coal quality is associated with more complicated co-occurrence network and elevated *Anaerostipes* abundance. Although it is necessary to conduct more studies to confirm their causality, these findings suggest that the bacterial community may be related to the coal quality and it may be possible to develop novel bacterial strategies for coal quality improvement.

## Data Availability

The original contributions presented in the study are included in the article/Supplementary material, further inquiries can be directed to the corresponding authors.

## References

[ref1] Borowski G. Ozga M. (2020). Comparison of the processing conditions and the properties of granules made from fly ash of lignite and coal. Waste Manag. 104, 192–197. doi: 10.1016/j.wasman.2020.01.024 31981820

[ref2] Bui T. de Vos W. M. Plugge C. M. (2014). Anaerostipes rhamnosivorans sp. Nov., a human intestinal, butyrate-forming bacterium. Int. J. Syst. Evol. Microbiol. 64, 787–793. doi: 10.1099/ijs.0.055061-0 24215821

[ref3] Dong X. Wang F. Guo L. Han T. Dong X. Hu X. (2024). The inhibitory effect of microbes on coal spontaneous combustion. Fuel 375:132658. doi: 10.1016/j.fuel.2024.132658

[ref4] Guo H. Liu R. Yu Z. Zhang H. Yun J. Li Y.. (2012). Pyrosequencing reveals the dominance of methylotrophic methanogenesis in a coal bed methane reservoir associated with eastern Ordos Basin in China. Int. J. Coal Geol. 93, 56–61. doi: 10.1016/j.coal.2012.01.014

[ref5] Hamidovic S. Cvijovic G. G. Waisi H. Zivotic L. Soja S. J. Raicevic V.. (2020). Response of microbial community composition in soils affected by coal mine exploitation. Environ. Monit. Assess. 192:364. doi: 10.1007/s10661-020-08305-2, PMID: 32409938

[ref6] Kadowaki R. Tanno H. Maeno S. Endo A. (2023). Spore-forming properties and enhanced oxygen tolerance of butyrate-producing Anaerostipes spp. Anaerobe 82:102752. doi: 10.1016/j.anaerobe.2023.102752 37301503

[ref7] Lee J. Y. Kang W. Shin N. R. Hyun D. W. Kim P. S. Kim H. S.. (2021). Anaerostipes hominis sp. Nov., a novel butyrate-producing bacteria isolated from faeces of a patient with Crohn's disease. Int. J. Syst. Evol. Microbiol. 71:129. doi: 10.1099/ijsem.0.005129, PMID: 34870576

[ref8] Li X. Chen X. Zhang F. Zhang M. Zhang Q. Jia S. (2020). Energy calculation and simulation of methane adsorbed by coal with different metamorphic grades. ACS Omega 5, 14976–14989. doi: 10.1021/acsomega.0c00462 32637771 PMC7330900

[ref9] Li S. Gao L. Wang J. Rong G. Cao Y. (2020). Enhancement of floatability of low-rank coal using oxidized paraffin soap. RSC Adv. 10, 15098–15106. doi: 10.1039/d0ra02361b 35495444 PMC9052298

[ref10] Lloyd M. K. Trembath-Reichert E. Dawson K. S. Feakins S. J. Mastalerz M. Orphan V. J.. (2021). Methoxyl stable isotopic constraints on the origins and limits of coal-bed methane. Science 374, 894–897. doi: 10.1126/science.abg0241, PMID: 34762461

[ref11] Morinaga K. Kusada H. Watanabe M. Tamaki H. (2021). Complete genome sequence of *Anaerostipes caccae* strain L1-92(T), a butyrate-producing bacterium isolated from human feces. Microbiol. Resour. Announc. 10:21. doi: 10.1128/MRA.00056-21, PMID: 33888495 PMC8063638

[ref12] Pan Y. Kang P. Hu J. Song N. (2021). Bacterial community demonstrates stronger network connectivity than fungal community in desert-grassland salt marsh. Sci. Total Environ. 798:149118. doi: 10.1016/j.scitotenv.2021.149118 34332392

[ref13] Park H. Wang L. Yun J. H. (2021). Coal beneficiation technology to reduce hazardous heavy metals in fly ash. J. Hazard. Mater. 416:125853. doi: 10.1016/j.jhazmat.2021.125853 34492803

[ref14] Robbins S. J. Evans P. N. Esterle J. S. Golding S. D. Tyson G. W. (2016). The effect of coal rank on biogenic methane potential and microbial composition. Int. J. Coal Geol. 154-155, 205–212. doi: 10.1016/j.coal.2016.01.001

[ref15] Ryan M. P. Pembroke J. T. (2018). Brevundimonas spp: emerging global opportunistic pathogens. Virulence 9, 480–493. doi: 10.1080/21505594.2017.1419116 29484917 PMC5955483

[ref16] Saha D. Roychowdhury T. (2023). Characterisation of coal and its combustion ash: recognition of environmental impact and remediation. Environ. Sci. Pollut. Res. 30, 37310–37320. doi: 10.1007/s11356-022-24864-y 36571687

[ref17] Shigeno Y. Kitahara M. Shime M. Benno Y. (2019). Phascolarctobacterium wakonense sp. Nov., isolated from common marmoset (*Callithrix jacchus*) faeces. Int. J. Syst. Evol. Microbiol. 69, 1941–1946. doi: 10.1099/ijsem.0.003407 31038451

[ref18] Sun L. Zhang H. Cao Y. Wang C. Zhao C. Wang H.. (2019). Fluoxetine ameliorates dysbiosis in a depression model induced by chronic unpredicted mild stress in mice. Int. J. Med. Sci. 16, 1260–1270. doi: 10.7150/ijms.37322, PMID: 31588192 PMC6775263

[ref19] Thomas G. Fariborz G. (2000). Effect of geological processes on coal quality and utilization potential: review with examples from western Canada. J. Hazard. Mater. 74, 109–124. doi: 10.1016/s0304-3894(99)00202-2, PMID: 10781721

[ref20] Tollefson J. (2017). World's carbon emissions set to spike by 2% in 2017. Nature 551:283. doi: 10.1038/nature.2017.22995, PMID: 29144478

[ref21] Wang B. Wang Y. Cui X. Zhang Y. Yu Z. (2019). Bioconversion of coal to methane by microbial communities from soil and from an opencast mine in the Xilingol grassland of Northeast China. Biotechnol. Biofuels 12:236. doi: 10.1186/s13068-019-1572-y 31624498 PMC6781394

[ref22] Watanabe Y. Nagai F. Morotomi M. (2012). Characterization of *Phascolarctobacterium succinatutens* sp. Nov., an asaccharolytic, succinate-utilizing bacterium isolated from human feces. Appl. Environ. Microbiol. 78, 511–518. doi: 10.1128/AEM.06035-11 22081579 PMC3255759

[ref23] Wu F. Guo X. Zhang J. Zhang M. Ou Z. Peng Y. (2017). *Phascolarctobacterium faecium* abundant colonization in human gastrointestinal tract. Exp. Ther. Med. 14, 3122–3126. doi: 10.3892/etm.2017.4878 28912861 PMC5585883

[ref24] Xu H. Xiao Q. Dai Y. Chen D. Zhang C. Jiang Y.. (2023). Selected Bacteria are critical for Karst River carbon sequestration via integrating multi-omics and hydrochemistry data. Microb. Ecol. 86, 3043–3056. doi: 10.1007/s00248-023-02307-6, PMID: 37831075

[ref25] Zhai Y. Liu X. Han J. Zou Y. Huang Y. Wang H.. (2022). Study on the removal characteristics of different air pollution control devices for condensable particulate matter in coal-fired power plants. Environ. Sci. Pollut. Res. 29, 34714–34724. doi: 10.1007/s11356-021-17952-y, PMID: 35040059

[ref26] Zhang Q. Wu Y. Wang J. Wu G. Long W. Xue Z.. (2016). Accelerated dysbiosis of gut microbiota during aggravation of DSS-induced colitis by a butyrate-producing bacterium. Sci. Rep. 6:27572. doi: 10.1038/srep27572, PMID: 27264309 PMC4893749

[ref27] Zhou Y. Lian Y. Liu T. Jin X. Wang Z. Liu X.. (2023). Impacts of high-quality coal mine drainage recycling for replenishment of aquatic ecosystems in arid regions of China: bacterial community responses. Environ. Res. 223:115083. doi: 10.1016/j.envres.2022.115083, PMID: 36529333

[ref28] Zhou Y. Zi T. Lang J. Huang D. Wei P. Chen D.. (2020). Impact of rural residential coal combustion on air pollution in Shandong, China. Chemosphere 260:127517. doi: 10.1016/j.chemosphere.2020.127517, PMID: 32758768

