# Supplementary material for: Distinct bacterial signature in the raw coal with different heating value
Source: Front Microbiol. 2024 Sep 5;15:1459596. doi: 10.3389/fmicb.2024.1459596 (PMC11410599; doi:10.3389/fmicb.2024.1459596)
Supplement: Supplementary file 1 [file Presentation_1.pdf]

Supplementary materials

Haijiang Zou[1,2#], Miaomiao Tian[3#], Jianmin Xu[2*], Guowei Li[4], Hui Chen[5], Junjun Yang[4], Pengtao Ling[4], Zhenxing Shen[1], Siyu Guo[6,7*]

[1] *Department of Environmental Science and Engineering, Xi'an Jiaotong University, Xi'an 710049, China*

[2] *Shaanxi Coalbed Methane Development Co., Shaanxi Coal and Chemical Industry Group Co., Ltd, Xi'an, Shaanxi, China,710000*

[3] *Center for Mitochondrial Biology and Medicine, The Key Laboratory of Biomedical Information Engineering of Ministry of Education, School of Life Science and Technology, Xi'an Jiaotong University, Xi'an, Shaanxi, China,710049*

[4] *Shaanxi Coal Industry Company Limited, Xi'an, Shaanxi, China,710000*

[5] *Xijing Hospital of Digestive Diseases, Xijing Hospital of Air Force Military Medical University, Xi'an, China*

[6] *Department of Civil Engineering, Xi'an Jiaotong University, Xi'an 710049, China*

[7]*School of Civil Engineering and Architecture, Henan University of Science and Technology, Luoyang 471000, China*

[#] These authors contributed equally to this work.

*Author to whom correspondence should be addressed.

Jianmin Xu

Email: Xujianmin_sxsm@hotmail.com

Shaanxi Coalbed Methane Development Co., Shaanxi Coal and Chemical Industry Group Co., Ltd, Xi'an, Shaanxi, China,710000

Siyu Guo

Email: tammy_lee@stu.xjtu.edu.cn

Department of Civil Engineering, Xi'an Jiaotong University, Xi'an 710049, China

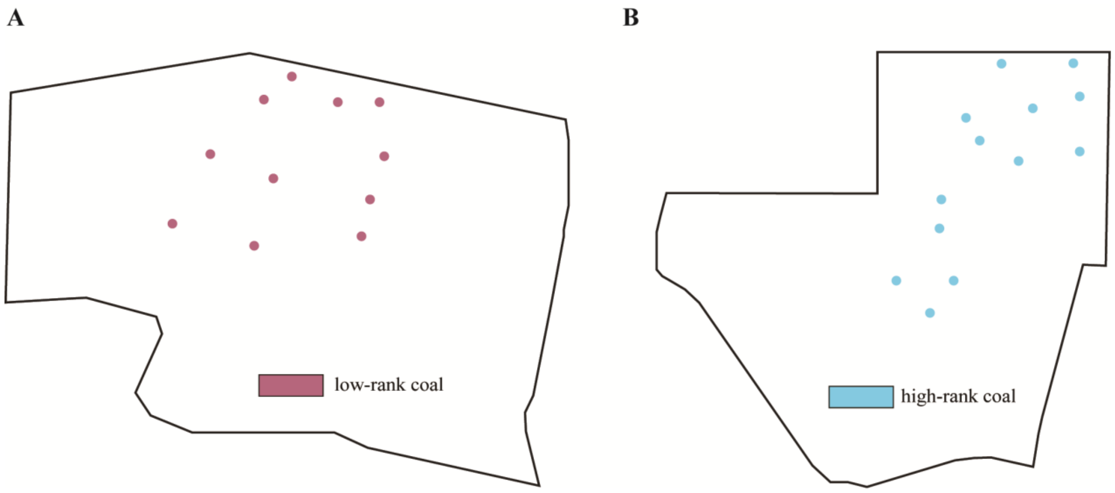

Supplementary Figure 1. Locations of coal samples

A. The locations of coal samples in the low-rank coal group; B. The locations of coal samples in the high-rank coal group

Engineering scale 1:10000

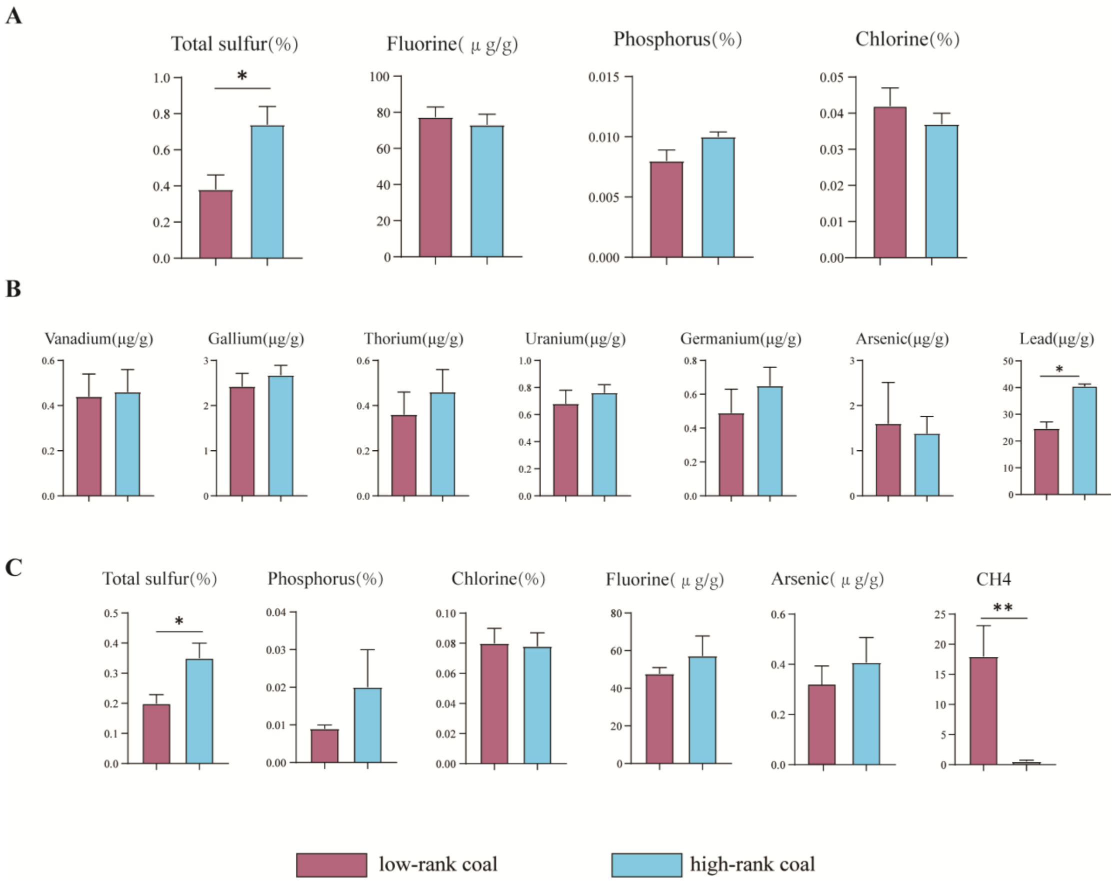

Supplementary Figure 2. Chemical elements and heavy metal concentrations in the raw coal and clean coal.

A. Chemical elements concentrations comparison in the raw coal; B. Heavy metal concentrations comparison in the raw coal; C. Chemical elements and heavy metal concentrations in the clean raw. * $P<0.05$
